# *Citrobacter braakii* Yield False-Positive Identification as *Salmonella*, a Note of Caution

**DOI:** 10.3390/foods10092177

**Published:** 2021-09-14

**Authors:** Joanna Pławińska-Czarnak, Karolina Wódz, Magdalena Kizerwetter-Świda, Tomasz Nowak, Janusz Bogdan, Piotr Kwieciński, Adam Kwieciński, Krzysztof Anusz

**Affiliations:** 1 Department of Food Hygiene and Public Health Protection, Institute of Veterinary Medicine, Warsaw University of Life Sciences, Nowoursynowska 159, 02-776 Warsaw, Poland; janusz_bogdan@sggw.edu.pl (J.B.); krzysztof_anusz@sggw.edu.pl (K.A.); 2 Laboratory of Molecular Biology, Vet-Lab Brudzew, Ul. Turkowska 58c, 62-720 Brudzew, Poland; karolina.wodz@labbrudzew.pl (K.W.); tomasz@labbrudzew.pl (T.N.); vetlab@interia.pl (P.K.); kwiecinski@vetlabbrudzew.pl (A.K.); 3 Department of Preclinical Sciences, Faculty of Veterinary Medicine, Warsaw University of Life Sciences-SGGW, Nowoursynowska 159, 02-776 Warsaw, Poland; magdalena_kizerwetter_swida@sggw.edu.pl

**Keywords:** *Salmonella* spp., *Citrobacter braakii*, meat, poultry, pork, beef

## Abstract

Background: Globally, *Salmonella enterica* is one of the leading causes of foodborne illness in humans. Food of animal origin is obligatorily tested for the presence of this pathogen. Unfortunately, in meat and meat products, this is often hampered by the presence of background microbiota, which may present as false-positive *Salmonella*. Methods: For the identification of *Salmonella* spp. from meat samples of beef, pork, and poultry, the authorized detection method is PN-EN ISO 6579-1:2017-04 with the White–Kauffmann–Le Minor scheme, two biochemical tests: API 20E and VITEK II, and a real-time PCR-based technique. Results: Out of 42 presumptive strains of *Salmonella*, 83.3% *Salmonella enterica* spp. *enterica*, 14.3% *Citrobacter braakii*, and 12.4% *Proteus mirabilis* were detected from 180 meat samples. Conclusions: Presumptive strains of *Salmonella* should be identified based on genotypic properties such as DNA-based methods. The aim of this study was the isolation and identification of *Salmonella* spp. from miscellaneous meat sorts: beef, pork, and poultry.

## 1. Introduction

*Salmonella* is one of the most important foodborne pathogens and a leading cause of foodborne illness in humans in the EU [1]. The source of the infection is usually contaminated food products of animal origin. Continuous surveillance of the occurrence of this pathogen in foods is required to ensure public health. Therefore, official food testing methodology according to European and national food legislation is necessary. Moreover, the crucial issues are the rapidity, efficiency, and accuracy of these methods. Traditionally, bacteriological culture methods have been used for isolation and identification of *Salmonella* spp. Colonies with a morphology indicative of *Salmonella* spp. are then selected for further studies, and identification is based on the determination of biochemical features and completed by serotyping. The specificity of meat samples makes it a challenging material for routine bacteriological testing as it contains a high number of indigenous microorganisms. Another complication may be the low *Salmonella* number compared to other bacteria in the sample. Enrichment culture procedures are aimed at increasing the salmonellae population but, at the same time, the number of background bacteria also increases. The reliable identification of *Salmonella* spp. is essential to ensure food safety. However, the presence of similarities in phenotypic characteristics between closely related bacteria may lead to incorrect identification. Bacteria belonging to the genus *Citrobacter* are particularly often incorrectly identified as *Salmonella* spp.

This study aimed to compare the identification of *Salmonella* spp. isolates obtained from meat samples using routine bacteriological methods and molecular biology methods.

## 2. Materials and Methods 

### 2.1. Sampling

A total of 180 raw meat samples (60 beef, 60 pork, and 60 poultry) were obtained from meat processing plants cutting beef, pork, and poultry carcasses in central Poland. All samples were derived from carcass parts classified by official veterinary inspections as fit for human consumption. All samples collected as a single sample weighed at least 200 g for each type of meat (from parts of carcasses for culinary use, i.e., roast beef, entrecote, boneless ribs, neck, breast, and thigh). The meat samples were collected randomly using the aseptic technique and were packed in separate sterile bags, which were then labeled. All samples were transported to the laboratory in refrigerated containers at a temperature of 4 °C within one hour of collection.

### 2.2. Salmonella spp. Isolation and Identification

*Salmonella* spp. from all samples were isolated in accordance with PN-EN ISO 6579-1:2017-04 Microbiology of the food chain—Horizontal method for the detection, enumeration and serotyping of *Salmonella*—Part 1: Detection of *Salmonella* spp. [2].

Samples were pre-enriched: for pork and beef samples, 10 g of each sample was mixed with 90 mL buffered peptone water (BPW GRASO, Starogard, Poland), and 25 g of each poultry sample was mixed with 225 mL BPW at a temperature of 25 °C (±3 °C) in sterile stomacher bags (Whirl-Pak, NAsco, Madison, WI, USA), placed in a stomacher and crushed for 2 min. The selective proliferation of *Salmonella* spp. was carried out using modified semi-solid Rappaport-Vassiliadis (MSRV) agar (GRASO, Starogard, Poland) and Muller–Kauffmann tetrathionate-novobiocin (MKTTn) broth (GRASO, Starogard, Poland). Two selective enrichment media, xylose lysine deoxycholate agar (XLD; GRASO, Starogard, Poland) and brilliant green agar (BGA; OXOID, Hampshire, United Kingdom), were used. *Salmonella* suspect colonies were transferred to a non-selective nutrient agar (GRASO, Starogard, Poland) to obtain the pure culture for further testing and a semi-solid medium by Garda for testing flagellar antigens. Serotyping was performed by slide agglutination with commercial H poly antisera for verification of the genus *Salmonella enterica* (IBSS Biomed, Kraków, Poland), O group antisera to determine O group (IBSS Biomed, Kraków, Poland), and H phase and H factor antisera to determine H phase and H factor (IBSS Biomed, Kraków, Poland, Bio-Rad, Hercules, CA, USA), according to the White–Kauffmann–Le Minor scheme. 

#### 2.2.1. Biochemical Strain Identification

Colonies showing morphology typical for *Salmonella* spp. on selective agars were subjected to biochemical identification using two commercially available tests: API 20E (BioMérieux, Craponne, France) and a VITEK2 COMPACT automated system for bacterial identification. VITEK^®^ 2 GN cards (BioMérieux, Craponne, France) with reference strains for *E. coli* ATCC 25922, *Salmonella* Typhimurium ATCC 14028, *Salmonella* Enteritidis ATCC 1307,6 and *Pseudomonas aeruginosa* ATCC 27853 served as a quality check. Both tests were used according to the manufacturer’s instructions.

#### 2.2.2. Confirmation of Salmonella Identification with Molecular Biology Methods

A real-time PCR method based on the detection of genes specific for *Salmonella* spp. was used to confirm presumptive identification. DNA for real-time PCR was extracted from bacterial cells using a commercial Kylt^®^ DNA Extraction-Mix II (Anicon, Emstek, Germany). For detection of *Salmonella* spp., a commercial Kylt^®^ *Salmonella* spp. (Anicon, Emstek, Germany) kit was used, and, for simultaneous detection of *Salmonella* Enteritidis and *Salmonella* Typhimurium, a commercial Spp-Se-St PCR (BioChek, Reeuwijk, The Netherland) kits was used. Both real-time PCR tests to detect *Salmonella* were performed according to the manufacturer’s instructions using an Applied Biosystems 7500 Fast Real-Time PCR System (Thermo, Waltham, MA, USA).

### 2.3. Antibiotic Resistance Test

Antimicrobial susceptibility was assessed by determining the MIC values using a VITEK^®^ 2 System and an AST-GN96 card for Gram-negative bacteria (BioMérieux). 

To analyze MIC patterns and detect phenotypes of *Citrobacter braakii*, an Advanced Expert System (AES, BioMérieux, Craponne, France) and VITEK^®^ 2 GN 96 cards (BioMérieux, Craponne, France) were used. The MICs were interpreted according to the Clinical and Laboratory Standards Institute (CLSI) and FDA breakpoints (CLSI M100-ED28, 2018).

### 2.4. Statistical Assessment

Statistical testing was performed with a Statistica 13.1 software package (StatSoft, Kraków, Poland). Descriptive statistics were computed to determine the proportions of isolates resistant to different antimicrobial agents. The chi-square test was adopted for the determination of the statistical significance of differences between the proportions.

## 3. Results

**Figure 1:**
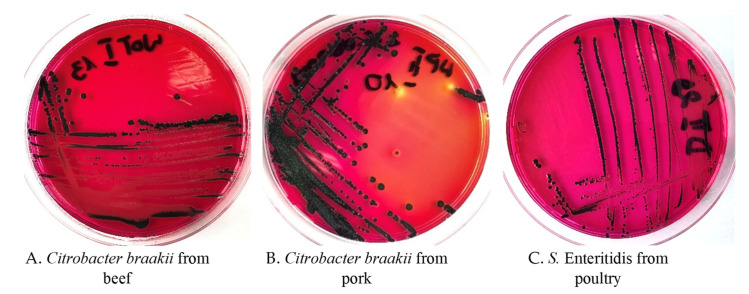
*Citrobacter braakii* from a meat sample of (**A**) beef and (**B**) pork, and *Salmonella* Enteritidis from a meat sample of (**C**) poultry, on XLD media.

The accuracy of identification obtained with the first biochemical test, API 20E (BioMérieux, Craponne, France), for some of the isolates of *Salmonella* like colonies on selective agars (Figure 1) was unsatisfactory, e.g., *Salmonella* spp. 71.9% and *Citrobacter freundii* 25% [3] Sero-diagnosis was difficult because it showed autoagglutinations or the test with the group sera was positive (especially DO), which could cause presumptive *Salmonella* diagnosis. Out of 180 meat samples, 23.33% indicated *Salmonella* spp. or presumptive strains of *Salmonella*. After obtaining the biochemical pattern from all 42 strains, 35 were confirmed as belonging to *Salmonella enterica* spp. *enterica*, 6 proved to be *Citrobacter braakii,* and 1 *Proteus mirabilis*. The results of the occurrence of *Salmonella* spp., *C. braakii*, and *Proteus mirabilis* in the meat samples tested are presented in Table 1.

The accuracy of identification obtained with the first biochemical test, API 20E (BioMérieux, Craponne, France), for some of the isolates of *Salmonella* like colonies on selective agars (Figure 1) was unsatisfactory, e.g., *Salmonella* spp. 71.9% and *Citrobacter freundii* 25% [3] Sero-diagnosis was difficult because it showed autoagglutinations or the test with the group sera was positive (especially DO), which could cause presumptive *Salmonella* diagnosis. Out of 180 meat samples, 23.33% indicated *Salmonella* spp. or presumptive strains of *Salmonella*. After obtaining the biochemical pattern from all 42 strains, 35 were confirmed as belonging to *Salmonella enterica* spp. *enterica*, 6 proved to be *Citrobacter braakii,* and 1 *Proteus mirabilis*. The results of the occurrence of *Salmonella* spp., *C. braakii*, and *Proteus mirabilis* in the meat samples tested are presented in Table 1.

General results based on colony morphology on selective agars, biochemical properties, and the PCR technique are presented in Table 2.

No *Salmonella* spp. were isolated from beef meat samples but three *Citrobacter braakii* were isolated. From pork meat samples, 1.67% (*n* = 1) was *Salmonella* spp. positive, *Citrobacter braakii* were isolated from three samples, and *Proteus mirabilis* from one. In poultry, 56.67% of samples (*n* = 35) isolated were *Salmonella* spp. Amongst isolated species, *Salmonella enterica subsp. enterica* was detected. The most common serovars were *S.* Enteritidis (55.88%, *n* = 19), *S.* Derby (14.71%, *n* = 5), and *S.* Newport (14.71%, *n* = 5); nd less frequently isolated were *S.* Infantis (5.88%, *n* = 2), *S.* Kentucky (5.88%, *n* = 2), and *S*. Mbandaka (2.94%, *n* = 1). 

The colonies formed by *Citrobacter braakii* and *Salmonella* Enteritidis on XLD selective media look almost identical (Figure 1).

The antibiotic resistance studies showed that all strains of *Citrobacter braakii* were susceptible to ampicillin, cephalosporins (III generation cefoperazone, ceftiofur, and IV generation cefquinome), aminoglycosides (gentamicin, neomycin), enrofloxacin, and trimethoprim/sulfamethoxazole, while resistance to other antibiotics was variable. For individual strains of *Citrobacter braakii* isolated from pork and beef samples, Table 3 presents several multi-drug resistance patterns.

## 4. Discussion

The phenotypic heterogeneity amongst bacteria is well-known, especially amongst closely related microorganisms. However, in the case of pathogens such as *Salmonella*, the misidentification may lead to a serious public health threat. In 2000, Manafi noticed that conventional media for the detection of *Salmonella* have a very poor specificity, creating an abundance of false positives such as *Citrobacter* or *Proteus* among the rare real positive *Salmonella* [4]. Twenty years later, despite many modifications and the development of further enriched media for the detection of *Salmonella*, there are still problems with the rapid identification of *Salmonella* spp. in meat. 

There is no information in the world literature about difficulties in interpreting the results obtained when the result is “presumably *Salmonella*”. However, it should be remembered that the experience of a laboratory technician and their proficiency in conducting research are crucial for the correctness of the conducted research. At the same time, the necessity to introduce molecular diagnostics for the final confirmation of samples with the result “presumably *Salmonella*” should be obligatory, not only to protect public health, but also in connection with the handling of meat on the EU market. Mistakenly qualifying *Citrobacter braakii* as *Salmonella* spp. may lead to the withdrawal from the market of meat or meat products and may cause risk of economic loss for farmers and producers. The requirements of the PN-EN ISO 6579-1: 2017-04 standard indicate that the result should specify the serotype of *Salmonella* spp. with the provisions of the Commission Regulation (EC) No/ 2073/2005 of 15 November 2005 on microbiological criteria for foodstuffs [2,5]. However, in the Rapid Alert System for Food and Feed (RASFF) system, sometimes an alert notification occurs with a risk description of “serious” resulting in withdrawal from the customer because apathogenic microorganisms identified as “no definition–*Salmonella*” in beef was detected [6].

Reliable identification of *Salmonella* is crucial for ensuring public health security. Culture-based methods, biochemical identification, and serotyping are traditionally used and recommended by relevant standards. However, considerable variability is observed in the biochemical properties of some *Salmonella, Proteus*, and *Citrobacter* isolates. The consequence of this is that some “presumptive *Salmonella*” isolates cannot be identified by subsequent serotyping. Previous studies on the reliability of the API 20E test showed both good and inaccurate results. The presumptive *Salmonella* identification was confirmed by API 20E, but 24% of the isolates were recognized as *Citrobacter* spp. and 16.4% as *Proetus* spp. [7]. The results presented in this study are in accordance with the above observations. We found that 16.7% of 42 isolates recognized as presumptive *Salmonella* were confirmed as *Citrobacter braakii*. 

Recently, many alternative non-culture methods have been described for *Salmonella* detection. These methods are based on DNA analysis or immunological reactions. A significant disadvantage of traditional methods is the time required for all culture procedures, which may take up to a few days for a presumptive identification. The available literature data indicate that the evident superiority of DNA-based methods is their high specificity and that results can be obtained after 24 h [8,9]. 

Bacteria belonging to the *Citrobacter* genus are closely related to *Salmonella*; thus, some similarities in cell surface antigens and biochemical properties occur between them. Pilar et al. proved that approximately one-third of *Citrobacter* and *Salmonella* genes are composed of core genes, confirming their close relationships [10]. This surprisingly high genotypic similarity can be explained by their common evolutionary history and genetic exchange [11]. All the properties of *Citrobacter* spp. listed above may result in false identification of these bacteria as *Salmonella*. It is also important to mention that it takes another day or two to confirm questionable identifications and, therefore, it takes longer to obtain the results and increases the costs of analysis.

Our results suggest that DNA-based methods should be used in cases of questionable results of *Salmonella* identification based on phenotypic properties.

On the other hand, further research on *C. braakii* is desirable in order to determine the current potential pathogenicity for humans, especially in view of the developing antibiotic resistance of *C. braakii*, which may contribute to the spread of resistance genes in the environment.

## Figures and Tables

**Table 1:** Incidence of *Salmonella* spp., *Citrobacter braakii*, and *Proteus mirabilis* in meat samples.

Type of Meat	All Samples	*Salmonella* spp.	*Citrobacter braakii*	*Proteus mirabilis*
beef	60	-	5% (*n* = 3)	-
pork	60	1.67% (*n* = 1)	5% (*n* = 3)	1.67% (*n* = 1)
poultry	60	56.67% (*n* = 34)	-	-

**Table 2:** Identification of *Salmonella* spp. isolates obtained from meat samples based on colony morphology on selective agars, biochemical properties, and the PCR technique.

Type of Meat	Number of Samples	Number of Isolates Forming *Salmonella*-like Colonies on Selective Agars.	Serological Tests			Confirmation of *Salmonella* spp. Identification Based on					
			Autoagglutination	Polyvalent HM	Group Antisera	Biochemical Properties					PCR Technique
						API		VITEK			
						*Salmonella* spp.	*Salmonella* spp./*Citobacter freundii*	*Salmonella enterica* ssp. *enterica*, *Salmonella* Enteritidis	*Proteus mirabilis*	*Citrobacter braakii*	*Salmonella* spp.
Beef	60	3	1	1	1	0	3	0	0	3	0
Pork	60	4	1	2	2	1	4	1	1	3	1
Poultry	60	35	6	29	29	35	0	35	0	0	35

**Table 3:** Antimicrobial resistance common to the variously identified *Citrobacter braakii* isolated from samples of pork and beef.

Type of Meat	*Citrobacter* sp.	Antimicrobial Resistance															
		AMP	AMX/CL	CFX	CFT	CFP	CFTI	CFQ	IMP	GEN	NEO	FLU	ENR	MRB	TET	FLR	TR/SMX
pork	*C. braakii*	S	S	R	R	S	S	S	R	S	S	S	S	S	S	I	S
pork	*C. braakii*	S	S	R	I	S	S	S	S	S	S	S	S	S	S	R	S
pork	*C. braakii*	S	S	I	S	S	S	S	S	S	S	S	I	S	S	R	S
beef	*C. braakii*	S	I	I	I	S	S	S	S	S	S	S	S	S	S	R	S
beef	*C. braakii*	S	S	I	R	S	S	S	I	S	S	S	S	S	S	I	S
beef	*C. braakii*	S	S	I	R	S	S	S	S	S	S	R	S	S	R	I	S

AMP—ampicillin, AMX/CL—amoxicillin and clavulanic acid, CFX—cephalexin, CFT—cephalothin, CFP—cefoperazone, CFTI—ceftiofur, CFQ—cefquinome, IMP–imipenem, GEN—gentamicin, NEO—neomycin, FLU—flumequine, ENR—enrofloxacin, MRB—marbofloxacin, TET—tetracycline, FLR—florfenicol, TR/SMX—trimethoprim—sulfamethoxazole. Labelling strains as resistant (R), intermediate (I), or susceptible (S) for a specific antimicrobial is indicated in the rows below each antimicrobial.

## Data Availability

The data presented in this study are available on request from the corresponding author. The data are not publicly available due to their containing information that could compromise the image of the meat processing plants.
